# Point of care ultrasound for assisting in needle aspiration of spontaneous pneumothorax in the pediatric emergency department: a case series

**DOI:** 10.1186/2036-7902-6-S1-A23

**Published:** 2014-01-31

**Authors:** C Ng, JW Tsung

**Affiliations:** 1 Department of Pediatrics, Bellevue Hospital Center/NYU School of Medicine, NY, USA; 2 Departments of Emergency Medicine and Pediatrics, Mount Sinai School of Medicine, NY, USA

## Background

There is controversy regarding needle aspiration for primary spontaneous pneumothorax (PSP), with contradictory recommendations between the American College of Chest Physicians consensus statement (2001) which suggests that needle aspiration has little place in the management of PSP, and the British Thoracic Society guidelines (2010) which recommend that needle aspiration be attempted first for all cases of PSP where drainage is deemed necessary. Studies have shown that there is no significant difference between needle aspiration and tube thoracostomy with regard to safety, rates of immediate success and early failure and has the advantages of decreasing pain, reducing rates of hospital admission and duration of hospital stay compared to tube thoracostomy. Point-of-care ultrasound can facilitate needle aspiration by decreasing the risk of complications and detect pneumothorax resolution during or reexpansion after the procedure.

## Patients and methods

This is a case series where the sonographic finding of the "lung point" on point-of-care ultrasound was used to facilitate needle aspiration to monitor pneumothorax resolution during or reexpansion after the procedure.

## Results

We report three cases of PSP in adolescents presenting to the pediatric ED, where needle aspiration was safely performed by using ultrasound to track the sonographic finding of the "lung point." This technique allows the determination of pneumothorax resolution or reexpansion in real-time.

## Conclusion

Point-of-care ultrasound may assist in the evaluation and management of spontaneous pneumothorax in the pediatric ED. Ultrasound assisted needle aspiration may be a safe and less painful option for pediatric ED patients with PSP.

